# Supplementary material for: Splice-Junction-Based Mapping of Alternative Isoforms in the Human Proteome
Source: Cell Rep. Author manuscript; Available in PMC 2020 Jan 15. (PMC6961840; doi:10.1016/j.celrep.2019.11.026)
Supplement: 3 [file NIHMS1546469-supplement-3.zip › DF2/PXD0010154/Lung-191-Q70EL1-TCYDSEDSGHLTDSECNQK.pdf]

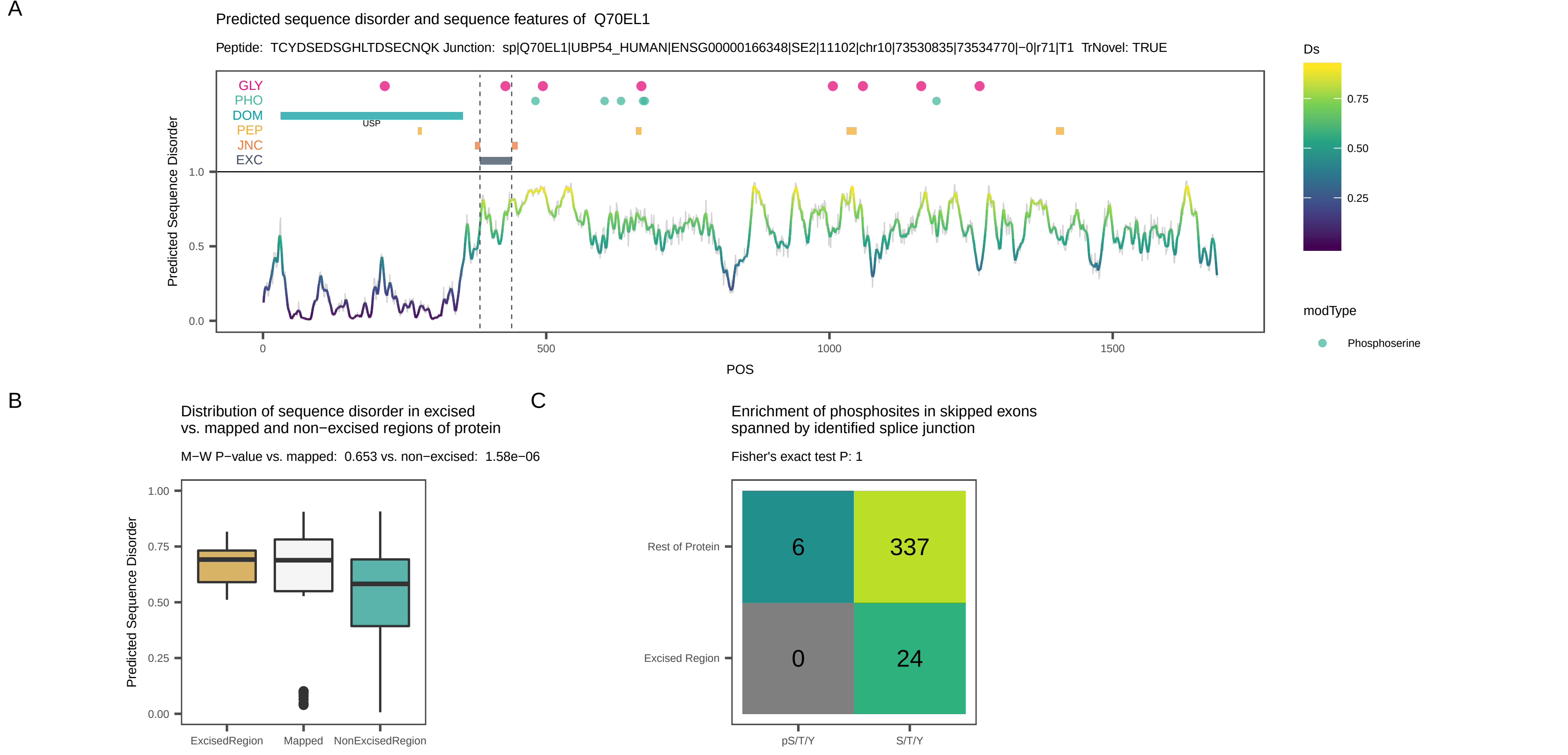

A
Predicted sequence disorder and sequence features of Q70EL1
Peptide: TCYDSEDSGHLTDSECNQK Junction: sp|Q70EL1|UBP54_HUMAN|ENSG00000166348|SE2|11102|chr10|73530835|73534770|−0|r71|T1 TrNovel: TRUE
GLY
PHO
DOM
PEP
JNC
EXC
USP
Predicted Sequence Disorder
1.0
0.5
0.0
0
500
1000
1500
POS
Ds
0.75
0.50
0.25
modType
Phosphoserine
B
Distribution of sequence disorder in excised vs. mapped and non−excised regions of protein
M−W P−value vs. mapped: 0.653 vs. non−excised: 1.58e−06
Predicted Sequence Disorder
1.00
0.75
0.50
0.25
0.00
ExcisedRegion
Mapped
NonExcisedRegion
C
Enrichment of phosphosites in skipped exons spanned by identified splice junction
Fisher's exact test P: 1
Rest of Protein
Excised Region
6
337
0
24
pS/T/Y
S/T/Y
